# Topical Janus kinase inhibitors in atopic dermatitis: a safety network meta-analysis

**DOI:** 10.1007/s11096-023-01569-x

**Published:** 2023-04-19

**Authors:** Carlos Alves, Ana Penedones, Diogo Mendes, Francisco Batel Marques

**Affiliations:** 1 Laboratory of Social Pharmacy and Public Health, Faculty of Pharmacy, University of Coimbra, Polo Ciencias da Saude, 3000-548 Coimbra, Portugal; 2 Clevidence, Lda., Taguspark, Oeiras, Portugal

**Keywords:** Atopic dermatitis, JAK inhibitors, Meta-analysis, Safety

## Abstract

**Background:**

Topical Janus kinase (JAK) inhibitors are being developed for the treatment of mild to moderate atopic dermatitis. However, comparative evidence on their safety profiles is still limited.

**Aim:**

This study aimed to compare the relative safety of topic JAK inhibitors in patients with atopic dermatitis.

**Method:**

Phase 2 and 3 clinical trials (RCTs) evaluating the efficacy and safety of topical JAK inhibitors in atopic dermatitis were searched on Medline, EMBASE and clinicaltrials.gov. The following outcomes were considered: any adverse event (AE), serious AEs, AEs leading to treatment discontinuation, any infection, any application site reaction.

**Results:**

Ten RCTs were included in this network meta-analysis. Tofacitinib was associated with a reduced risk of any AE when compared with ruxolitinib (OR 0.18, 95% CrI 0.03–0.92). The analyses for the remaining outcomes did not identify other statistically significant risk differences between the topical JAK inhibitors.

**Conclusion:**

Although tofacitinib seems to present a reduced risk of any adverse event compared with ruxolitinib, this was the only statistically significant result found between JAK inhibitors. Therefore, such findings should be interpreted with caution considering the scarce data available and the heterogeneity between the studies, and there is no robust evidence allowing pointing out clinically important differences between the safety profiles of the existing topical JAK inhibitors. Further pharmacovigilance activities are needed to confirm the safety profile of these drugs.

**Supplementary Information:**

The online version contains supplementary material available at 10.1007/s11096-023-01569-x.

## Impact statements

- Most of the adverse drug reactions associated with topical JAK inhibitors are expected, localized and non-serious events. However, it is relevant to perform comprehensive comparison of the safety profile of these new drugs, given the absence of head-to-head studies.
- Apart from a reduced risk of any adverse event associated with tofacitinib when compared with ruxolitinib, no other risk differences between JAK inhibitors have been identified.
- However, scarse data, between-studies heterogeneity and some methodological quality concerns with some studies preclude reaching definitive conclusions, with further studies being needed.

## Introduction

Atopic dermatitis is an inflammatory, relapsing skin disease affecting nearly 10% of adults and 20% of children [1]. Most cases of this disease are considered mild, but ≤ 10% of patients have severe skin lesions [1]. Intense pruritus, erythematous papules and epidermal hyperplasia characterize the lesions during the acute phase [2]. Chronic injuries from atopic dermatitis are characterized by hyperkeratosis, which may result in infections, insomnia, and reduced quality of life [2].

Patients with atopic dermatitis who do not respond to good skin care and emollients should begin treatment with topical corticosteroids and calcineurin inhibitors [1, 3]. These drugs have shown efficacy in the management of acute and chronic lesions and are recommended for use in active inflammatory disease and in the prevention of relapses [1, 3]. However, topically administered corticosteroids and calcineurin inhibitors are associated with safety concerns (e.g., skin atrophy, telangiectasia, and symptoms of skin irritation) that have triggered the development of new pharmacological alternatives [4, 5].

Both oral and topical pharmaceutical formulations of Janus kinase (JAK) inhibitors have been recently introduced to treat atopic dermatitis. Topical JAK inhibitors aim to improve drug delivery to the skin areas affected by the disease, accelerating the onset of action and reducing the risk of serious adverse drug reactions usually associated with oral formulations [5]. The first drug of this class to be approved was ruxolitinib, which is indicated for mild to moderate atopic dermatitis, when the disease is not adequately controlled with other topical options [6]. According to the results of clinical trials (RCTs), topical JAK inhibitors lessen pruritus, reduce pain and eczema, and improve sleeping [4, 5].

Most common, expected adverse reactions of topical JAK inhibitors are localized and non-serious. However, serious, clinically relevant, potential risks are associated with JAK inhibitors, usually occurring with oral formulations. For example, serious opportunistic infections, major adverse cardiac events (MACE), thromboembolic events, and malignancies have been observed [7, 8]. There are systematic reviews and pair-wise meta-analyses of RCTs that have evaluated the safety of topical JAK inhibitors in atopic dermatitis [9–11]. However, unlike network meta-analyses, those pair-wise meta-analyses did not establish indirect adjusted treatment comparisons between JAK inhibitors, offering little help to support informed decisions. Although there is one network meta-analysis compared the safety of topical JAK and phosphodiesterase inhibitor-4 inhibitors in patients with atopic dermatitis, data one safety profile comparisons are very limited since only one outcome was assessed, namely the incidence of treatment-related adverse events [12]. Given the absence of head-to-head studies from both experimental and observational nature, it might be useful to resort to network meta-analysis to perform a further evaluation of the safety profile of these novel topical pharmaceutical formulations, assessing the risk of additional overall and specific outcomes which have not been addressed in previous publications.

### Aim

This systematic review and network meta-analysis aimed to compare the relative safety of existing topical JAK inhibitors when used in the treatment of patients with atopic dermatitis.

## Method

The recommendations of the Centre for Reviews and Dissemination’s guidance for undertaking reviews in healthcare and the PRISMA extension statement for reporting systematic reviews incorporating network meta-analyses of health care interventions were followed in order to conduct and report this systematic review (Table S1) [13, 14]. The study was registered at PROSPERO (CRD42022329007) and at European Network of Centres for Pharmacoepidemiology and Pharmacovigilance (ENCePP) (EUPAS46966).

### Eligibility criteria

The following criteria were considered when selecting the studies for inclusion:

- Study design: randomized controlled trials (RCTs) of phase II and III;
- Population: patients with atopic dermatitis according to one of following diagnostic criteria: American Academic of Dermatology; Hanifin and Rjaka; Japanese Dermatological Association [15–17];
- Intervention: studies evaluating topical JAK inhibitors (delgocitinib, tofacitinib, ruxolinitib) used in the treatment of atopic dermatitis;
- Comparators: studies using placebo, active treatment or no treatment were considered;
- Outcomes: any adverse event; serious adverse events (i.e., any untoward medical occurrence that at any dose may result in death, threats life, require hospital admission or prolongation of existing hospital stay, or result in persistent or significant disability or incapacity); adverse events leading to treatment discontinuation; any infection; any application site reaction.
- Timing: no restrictions were applied to the length of follow-up;
- Language: only studies reported in English language were included.

### Information sources

Medline and EMBASE (using Ovid), and ClinicalTrials.gov were searched from their inception until August 11, 2022. A search by hand of the references lists of all relevant studies, systematic reviews and meta-analyses was performed to identify additional studies that could be elected for inclusion.

### Search strategy

Atopic dermatitis and drugs names, including the thesaurus terms and the International Nonproprietary Names (INN), were considered for search terms. No language filters were applied. Table S2 described the search strategy.

### Study records

The titles and abstracts were screened by hand by two independent researchers to select full articles for inclusion, as the established prespecified eligibility criteria. Disagreements were resolved by discussion and consensus with a third researcher.

### Data items

The subsequent data were extracted from the selected studies: reference, publication year, RCT phase (II or III), sample sizes, follow-up length, intervention (name, dosage, frequency, and duration of treatment), comparators and data on the safety outcomes (any adverse event, serious adverse events, adverse events leading to treatment discontinuation, any infection, and any application site reaction). Two researchers independently extracted data from each included study into a pre-designed form.

### Risk of bias of the individual studies

The risk of bias of each individual study was assessed using the “RoB 2 tool: A revised Cochrane risk of bias tool for randomized trials” [18]. Two major characteristics were considered when addressing the value of RCT on reporting adverse effects: the rigor of monitoring for the adverse effects during the study, and the completeness of reporting.

### Data synthesis

The Bayesian Markov Chain Monte-Carlo (MCMC) sampling in WinBUGS, version 1.4.3 (MRC Biostatistics Unit, Cambridge, UK) was used to perform a bayesian network meta-analysis [19]. 10,000 iterations were used to achieve convergence. A further 50,000 iterations were run on three chains, providing posterior sample of 150,000 values, on which the results of study were based [20]. Gelman-Rubin statistics and monitoring of the Monte Carlo error were used to assess the convergence of the models. The model fit for each outcome measure was assessed through the residual deviance and the deviance information criterion [21–23]. Odds Ratios (OR) described with 95% credible intervals were chosen as effect size measures for the Bayesian network meta-analyses. The following non-informative prior distributions were initially proposed: uniform (0.2) for standard deviation of the random effects model and normal (0, tau = 0.0001) for log[OR]s. However, for outcome measures characterized by sparse data (0 events observed in one or both arms), a strong prior was used: uniform (− 2.10;1.58^2^) for standard deviation of the random effects model and normal (− 2.34, tau = 1.62^2^) [24, 25]. The probability of each drug being the best (safest) among all drugs for each outcome measure was estimated by ranking the relative safety of all treatments [20]. R statistical software (R version 4.1.2) was used to perform network maps.

## Results

**Fig. 1:**
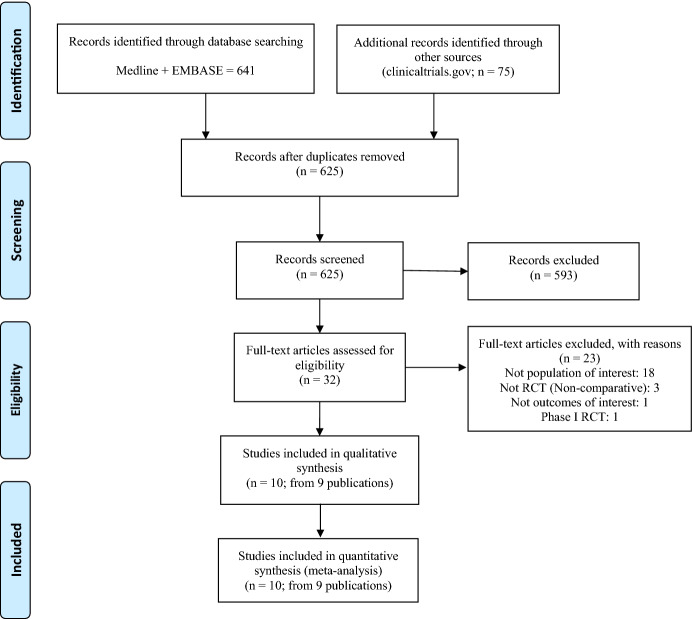
PRISMA flow diagram. From: Moher D, Liberati A, Tetzlaff J, Altman DG, The PRISMA Group (2009). Preferred Reporting Items for Systematic Reviews and Meta-Analyses: The PRISMA Statement. PLoS Med 6(7): e1000097. doi:10.1371/journal.pmed1000097. For more information, visit www.prisma-statement.org

The flow diagram of the search strategy is illustrated in Fig. 1. The search of the electronic databases returned 716 references. Ten studies were included after duplicates and studies with inadequate design have been excluded following the predefined criteria [4, 5, 26–32]. One publication reported the findings of two studies [5]. Table 1 describes the characteristics of the studies (design and time of follow-up, participants’ demographics, drugs, and sample sizes). Only two studies included patients using topical alternatives as background therapy. All studies used vehicle ointment (placebo) as comparator, but two studies used tacrolimus ointment and triamcinolone acetonide cream (TAC) as reference comparators [28, 31]. Table S3 described the frequency of the adverse events included in this network meta-analysis.

**Table 1:** Characteristics of the studies included

References	Year of publication	RCT phase	Region	Background therapy	Intervention			Comparator		Follow-up (weeks)
					Name	Dosage/ Frequency	Sample size (N)	Name	Sample size (N)	
Nakagawa et al. 2020 [4]	2020	III	Japan	None	Delgocitinib	0.5%, b.i.d	106	Vehicle ointment	52	4
Papp et al. 2021 [5] (TRuE-AD1)	2021	III	Global	None	Ruxolitinib	0.75%, b.i.d	300	Vehicle ointment	126	8
						1.5%, b.i.d	300			
Papp et al. 2021 [5] (TRuE-AD2)	2021	III	Global	None	Ruxolitinib	0.75%, b.i.d	298	Vehicle ointment	124	8
						1.5%, b.i.d	301			
Nakagawa et al. 2021 [26]	2021	III	Japan	None	Delgocitinib	0.25%, b.i.d	69	Vehicle ointment	68	4
Nakagawa et al. 2019 [27]	2019	II	Japan	None	Delgocitinib	0.25%, b.i.d	34	Vehicle ointment	35	4
						0.5%, b.i.d	34			
Nakagawa et al. 2018 [28]	2018	II	Japan	None	Delgocitinib	0.25%, b.i.d	69	Tacrolimus 0.1%, b.i.d Vehicle ointment	30	4
						0.5%, b.i.d	65			
						1%, b.i.d	66			
						3%, b.i.d	65		32	
NCT03725722 [29]	2018	II	USA, Australia, Canada	None	Delgocitinib	0.1%, b.i.d	49	Vehicle ointment	50	10
						0.3%, b.i.d	50			
						0.8%, b.i.d	50			
						2%, b.i.d	51			
NCT03683719 [30]	2018	II	USA, Denmark, Germany	None	Delgocitinib	0.1%, b.i.d	52	Vehicle ointment	50	18
						0.3%, b.i.d	51			
						0.8%, b.i.d	52			
						2%, b.i.d	53			
Kim et al. 2020 [31]	2020	II	USA, Canada	Topical (nonmedicated emollients), 2.5% hydrocortisone cream	Ruxolitinib	0.15%, q.d	51	Triamcinolone acetonide (TAC) 0.1%, b.i.d Vehicle ointment	51	8
						0.5%, q.d	51			
						1.5%, q.d	51			
						1.5%, b.i.d	50		52	
Bissonnette et al. 2016 [32]	2016	II	Canada	Topical (nonmedicated emollients)	Tofacitinib	2%, b.i.d		Vehicle ointment	34	4

RCT, randomized clinical trial; b.i.d., twice daily; q.d., once daily; USA, United States of America

Figure S1 shows the assessment of the risk of bias of the included studies: 7 RCTs were associated with concerns and 3 with a high risk of bias. The main methodological insufficiencies of the studies were the absence of information regarding the allocation concealment/randomization method, the monitoring process of the adverse events and the lack of detailed pre-specified data analysis plans.

### Network maps

Figure S2 depicts the network maps of the network meta-analyses. The thickness of the line connecting two treatments is proportional to the number of studies established the correspondent comparison.

### Any adverse event

Compared with placebo, tacrolimus (Odds Ratio [OR] 3.88, 95% credible interval [CrI] 1.04–15.02) increase the risk of any adverse event (Figure S3). Tacolimus also has an increased risk of any adverse event when compared to tofacitinib (OR 11.08, 95% CrI 1.56–83.72). Tofacitinib presents a reduced risk of any adverse event when compared with ruxolitinib (OR 0.18, 95% CrI 0.03–0.92). According to the ranking, tofacitinib is probably the safest treatment, followed by placebo and delgocitinib (Table S4).

### Serious adverse events

**Fig. 2:**
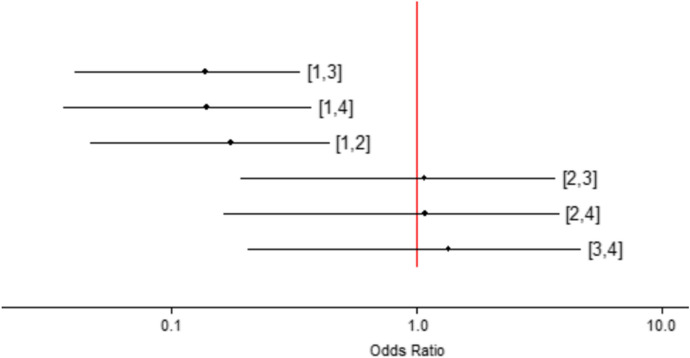
Risk of serious adverse events between tretaments. Legend: 1, Placebo; 2, ruxolitinib, 3, delgocitinib, 4, triamcinolone acetonide cream (TAC)

All treatments reduce the risk of serious adverse events versus placebo (Fig. 2). No further differences with statistical significancy were identified between treatments. TAC is the safest treatment, followed by delgocitinib and ruxolitinib (Table S4).

### Adverse events leading to treatment discontinuation

**Fig. 3:**
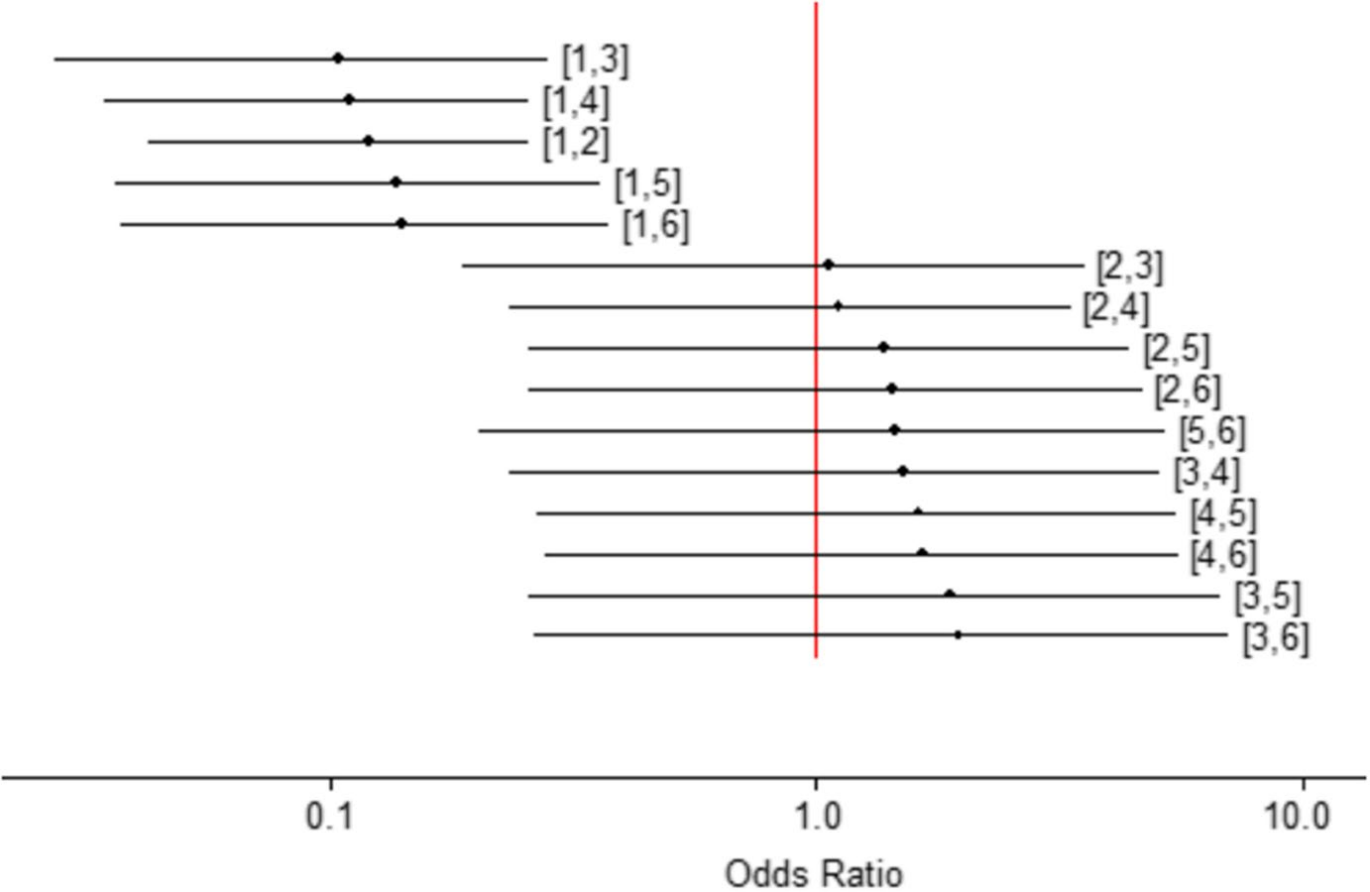
Risk of adverse events leading to treatment discontinuation. Legend: 1, Placebo; 2, ruxolitinib; 3, tofacitinib; 4, delgocitinib; 5, triamcinolone acetonide cream (TAC); 6, tacrolimus

The risk of adverse events leading to discontinuation is lower with every treatment than with placebo (Fig. 3). No additional risk differences were identified between treatments. Tofacitinib was the safest treatment, followed by delgocitinib and ruxolitinib (Table S4).

### Any infection

Compared with ruxolitinib, TAC is associated with a lower risk of any infection (OR 0.04, 95% CrI 0–0.52) (Figure S4). According to the ranking, TAC is the safest treatment, followed by placebo and tacrolimus (Table S4).

### Any application site reactions

All treatments present a reduced risk of any application site reactions compared with placebo (Figure S5). TAC is the safest treatment, followed by delgocitinib and ruxolitinib (Table S4).

### Model fit, model consistency, heterogeneity, and convergence

There may exist some lack of model fit for the predictions of the network meta-analyses of serious adverse events and any application site reaction, since the posterior mean residual deviances (*Dres*) of each of them (29.22 and 24.50, respectively) are greater than the number of unconstrained data points (22 and 16, respectively) (Table S5). The *Dres* estimates of the remaining network meta-analysis suggest that the model predictions fit well to the data. The contribution of the individual RCTs to the residual deviance is illustrated in Figure S6. Nakagawa and colleagues (2018), a 4 weeks, phase II RCT, which evaluates delgocitinib, emerges from the sample of studies as the one contributing largely for the residual deviance among the adverse events leading to treatment discontinuation meta-analysis [28].

The statistical analysis identified the presence of between-studies heterogeneity (σ) in these meta-analyses, once the results are different from zero (0), with the confidence intervals being slightly widened (Table S5). The estimates for the posterior mean deviances (*Dmodel*) and for the Deviance Information Criteria (*DIC*) indicate that the random effects model is the most adequate to conduct the analyses (Table S5).

Convergence seems to be achieved in all network meta-analyses, but difficulties were observed for the nodes including tacrolimus (treatment 6) in the any infection meta-analysis, according to the Gelman-Rubin statistics (Figure S7). In that case, the plots tended to stabilize just around iteration 70,000 for most of the comparisons (*R* < 1.05). The major reason for this may be that only one study of tacrolimus was included, and the respective branch has a reduced sample size (n = 30). Still, the Monte-Carlo standard error (MC error) analysis was estimated as < 5% of the posterior standard deviation (sd), suggesting that sufficient posterior samples have been used for inference (Table S6).

## Discussion

The activation of the JAK-STAT pathway is linked with the pathophysiology of atopic dermatitis. The cascade is crucial for the immune dysregulation that occurs, which promotes a response from Th2 cell and eosinophils, upregulates epidermal chemokines, pro-inflammatory cytokines, and pro-angiogenic factors and downregulates the function of the skin barrier [33]. JAK inhibitors are effective in the treatment of atopic dermatitis, since they inhibit the JAK-STAT pathway, resulting in an immunosuppressive activity [4, 5, 32]. Although uncommon, patients with atopic dermatitis receiving topical JAK inhibitors may experience serious adverse effects [6, 34].

Individuals with atopic dermatitis seem to be at an increased baseline risk of adverse drug reactions. The defects in the skin barrier and the disfunctions in the immune system caused by the disease predispose patients to suffer both topical and systemic secondary infections, with some rare cases being life-threatening [35–37]. Furthermore, evidence also suggests that patients with atopy are at an increased risk of experiencing more severe adverse effects [38]. Hence, it is clinically relevant to conduct comparisons between the safety profiles of topical JAK inhibitors that may contribute to improve the decision-making process in a high burden disease.

According to the results of this systematic review and network meta-analysis, topical JAK inhibitors appear to have comparable safety profiles. Only a comparison between two JAK inhibitors returned a statistically significant difference—the risk of any adverse event was lower with tofacitinib than with ruxolitinib. No further risk differences were found between the topical JAK inhibitors on either overall or specific safety outcomes. Nonetheless, it should be considered that only one study of tofacitinib was included in this network meta-analysis, which may create difficulties in comparing the safety profile of this drug and the remaining topical JAK inhibitors. Therefore, the identified statistically significant result mentioned before should be interpreted with caution, as well as the ranking place of tofacitinib when compared with other JAK inhibitors. Moreover, that the current network meta-analysis was not designed to perform comparisons between topical JAK inhibitors and other drugs used for atopic dermatitis, namely TAC or tacrolimus. The literature search strategy was designed to identify only the RCTs that evaluated topical JAK inhibitors, and the networks that have been established in this meta-analysis don’t include RCTs of other drugs. For this reason, the results found between topical JAK inhibitors and TAC or tacrolimus lack statistical robustness.

This network meta-analysis did not include systemic JAK inhibitors because these formulations are indicated for patients with a more severe condition, and they are not candidates to receive topical JAK inhibitors [39]. Also, the frequency of adverse events is higher in patients under treatment with systemic drugs, and it would not be methodologically appropriate to compare the safety profile of all JAK inhibitors in the same network meta-analysis [40–42].

As far as our knowledge is concerned, this is the first network-meta-analysis comparing the safety profile of topical JAK inhibitors based on data from several outcomes. There are other meta-analyses evaluating the safety of JAK inhibitors published in the scientific literature [9–11]. However, those studies contribute little to the relative safety comparison of topical JAK inhibitors, since they established indirect, unadjusted comparisons between treatments. And although there is a network-meta-analysis comparing topical JAK inhibitors, such study only evaluates the risk of treatment-related adverse events [12]. A strength of this work is the evaluation of safety outcomes different from those commonly addressed in published systematic reviews. Serious adverse events, infections, and application site reactions were specifically addressed in this network meta-analysis, constituting new useful safety information that can be taken into consideration to support clinical decision process when choosing a topical JAK inhibitor.

One of the main limitations of this network meta-analysis is that the frequency of events evaluated is rare. Excluding any adverse events and any infections, other events occur with low incidence and some RCTs reported zero cases; even application site reactions occurred with a modest frequency. In the sample of RCTs included in this network meta-analysis, very few cases of serious, systemic, opportunistic infections were reported, irrespectively of the treatment given to patients. Although there is evidence that patients with severe atopic eczema are at increased risk of cardiovascular disease, only two cases of major adverse cardiovascular events (one with TAC, one with ruxolitinib) were identified in the RCTs [43–45]. Both cases were judged as not related with treatments. A low frequency of such adverse events had already been noted in RCTs with systemic JAK inhibitors. Therefore, it was not possible to create further networks of studies to compare the risk of other adverse events between treatments, namely specific types of opportunistic infections, or cardiovascular events. Although real-world data may significantly contribute to perform a more comprehensive assessment of adverse drug reactions, no comparative, observational studies evaluating the risk of adverse events have been published in the scientific literature yet. Thus, they could not be considered in this analysis.

The results of the network meta-analysis should be interpreted in the light of some additional limitations. First, it was not possible to perform sensitivity analyses to assess the robustness of the results because of the rarity of the adverse events; for instance, it was not possible to form study networks to disaggregate data according to predefined variables (e.g., methodological quality scores of studies, background therapy). Second, the risk of bias was present in all studies (3 out of 10 were judged as having high risk of bias), namely because of insufficient information about allocation concealment or randomization procedures, adverse events monitoring, and data analysis plans in included studies. The protocols of the RCTs were not always available for public consultation, preventing investigators of carrying out a thorough assessment of the methodological quality of the studies. Third, the between-studies heterogeneity in the network meta-analyses. There are differences between studies regarding concomitant background therapies, durations of follow-up (from 4 to 18 weeks), sample sizes (from tents to hundreds of patients), and locations of recruitment sites. Such differences may comprise the causes of the heterogeneity. Fourth, it was identified some lack of model fit of the predictions from the network meta-analyses models used to analyse the risk of serious the adverse events and the risk of any application site reaction outcomes. This limitation was expected given the low rate of such events, as these networks included studies with low sample size, particularly phase II RCTs.

## Conclusion

Among all the analyses which have been conducted, only one statistically significant difference was identified between tofacitinib and ruxolitinib, with the former presenting a reduced risk of any adverse event. However, these findings should be interpreted with caution considering the scarce data available and the heterogeneity between the studies. Therefore, there is no robust evidence allowing pointing out clinically important differences between the safety profiles of the existing topical JAK inhibitors. Pharmacovigilance activities will be important to investigate and confirm potential adverse effects associated with this new class of drugs.

## Supplementary Information

Below is the link to the electronic supplementary material.

Supplementary file1 (DOCX 3342 KB)
